# Population coding and self-organized ring attractors in recurrent neural networks for continuous variable integration

**DOI:** 10.3389/fnetp.2025.1693772

**Published:** 2025-10-31

**Authors:** Roman Kononov, Vasilii Tiselko, Oleg Maslennikov, Vladimir Nekorkin

**Affiliations:** ^1^Nonlinear Dynamics Department, Gaponov-Grekhov Institute of Applied Physics of the Russian Academy of Sciences, Nizhny Novgorod, Russia; ^2^ Faculty of Radiophysics, Lobachevsky State University of Nizhny Novgorod, Nizhny Novgorod, Russia; ^3^ Center for Neurophysics and Neuromorphic Technologies, Moscow, Russia; ^4^ Phystech School of Applied Mathematics and Computer Science, Moscow Institute of Physics and Technology, Dolgoprudny, Moscow Region, Russia

**Keywords:** recurrent neural networks, bump attractors, population coding, continuous variable integration, nonlinear dynamics, network physiology, neural representation

## Abstract

Representing and integrating continuous variables is a fundamental capability of the brain, often relying on ring attractor circuits that maintain a persistent bump of activity. To investigate how such structures can self-organize, we trained a recurrent neural network (RNN) on a ring-based path integration task using population-coded velocity inputs. The network autonomously developed a modular architecture: one subpopulation formed a stable ring attractor to maintain the integrated position, while a second, distinct subpopulation organized into a dissipative control unit that translates velocity into directional signals. Furthermore, systematic perturbations revealed that the precise topological alignment between these modules is essential for reliable integration. Our findings illustrate how functional specialization and biologically plausible representations can emerge from a general learning objective, offering insights into neural self-organization and providing a framework for designing more interpretable and robust neuromorphic systems for navigation and control.

## 1 Introduction

A central challenge in physiology is to uncover how complex neural and physiological systems achieve robust, flexible information processing through the structured interaction of distributed components—a phenomenon deeply rooted in the principles of self-organization. In recent years, the rapidly growing field of network physiology has emphasized understanding the coordinated dynamics and functional connectivity within and across distinct subsystems, with the goal of elucidating mechanisms underlying adaptive behavior, resilience, and nonequilibrium phase transitions in living systems (Bartsch et al., 2015; Ivanov and Bartsch, 2014). Neural networks, in particular, serve as canonical models of such emergent dynamics, in which collective behaviors—ranging from oscillations to discrete or continuous attractor states—arise from recurring patterns of connectivity and population-level coding (Vladimirovich Maslennikov et al., 2022).

Within this framework, population coding and attractor dynamics have been recognized as fundamental organizing principles that underpin neural computation (Haken, 1983). Paradigmatic examples include bump and ring attractor networks, which enable the representation and integration of continuous variables. These networks are not confined to a single species or brain region but represent a species-agnostic neural motif for computation, found in contexts ranging from spatial orientation in mammals (Zhang, , 1996; Moser et al., 2008) to the internal compass of insects like the fruit fly (Kim et al., 2017), birds (Ben-Yishay et al., 2021), and even fish (Vinepinsky et al., 2020). This evolutionary convergence implies that by studying these motifs, we can gain insight into the general physiological principles of brain function across diverse species (Khona and Fiete, 2022; Basu et al., 2024). These distributed representations also exemplify physiological robustness, permitting reliable encoding in the presence of noise and fluctuating inputs (Averbeck et al., 2006; Pouget et al., 2000). In the hippocampal-entorhinal circuit, for instance, place and grid cells collectively generate a dynamic map of the environment (Hafting et al., 2005; McNaughton et al., 2006). Theoretically, such 2D spatial representations can be constructed by combining multiple 1D ring attractors, each encoding orientation along a different axis, highlighting the role of the ring attractor as a fundamental computational building block (Bush and Burgess, 2014).

Crucially, network-based models such as ring attractors not only capture these population-level coding schemes, but also provide a theoretical framework—rooted in the self-organization of collective variables—for understanding how internal states can be flexibly updated, maintained, and read out by downstream systems (Skaggs et al., 1994; Fiete et al., 2008; Seeholzer et al., 2019). In head direction systems and cortical integration circuits, the spontaneous emergence of ring attractors as solutions to path integration and spatial memory tasks exemplifies how nonequilibrium transitions and bifurcations in network structure give rise to functionally specialized modules (Georgopoulos et al., 1986; Salinas and Abbott, 1994; Ganguli and Sompolinsky, 2012; Knierim et al., 1995; Heinze et al., 2018). Such processes underscore a general principle, highlighted in the Synergetics tradition: physiological networks can self-organize connectivity patterns and explicit coding strategies to achieve both specialization and adaptive coordination among functional subunits.

Despite considerable progress, a central open question in both physiology and artificial intelligence remains: How can such modular, interpretable architectures—capable of robust continuous integration and flexibly dealing with circular variables—arise autonomously through learning mechanisms? Here, we use the term “autonomously” to describe the emergence of structured connectivity and dynamics driven by a high-level functional objective, rather than through handcrafted design. And how do the emergent patterns of organization constrain or enhance physiological function? Building bridges between biological plausibility and artificial network design is thus essential for advancing our theoretical understanding and for informing translational neuromorphic engineering (Banino et al., 2018; Izzo et al., 2023; Ganguli and Sompolinsky, 2012).

Motivated by these themes, and inspired by the pioneering insights of Hermann Haken, we here examine how a recurrent neural network trained with explicit population coding can self-organize into functional, physiologically congruent subpopulations that support robust, interpretable continuous variable integration. By dissecting the network’s emergent architecture, dynamical coding strategies, and the response to systematic perturbations, we aim to illuminate general organizing principles that underlie both neural computation and the broader dynamics of self-organized physiological networks.

## 2 Materials and methods

We address the problem of continuous navigation on a ring—a canonical task in theoretical and systems neuroscience—where an agent receives, at each timestep t , a velocity signal $v_{t}\in \left[-1,1\right]$ as input and must integrate it over time to estimate its angular position $x_{t}\in \left.\left[0,2\pi \right.\right)$ on the ring. The velocity input $v_{t}$ is generated synthetically using a diverse range of motion profiles, which ensures robust training and testing of the network’s ability to perform under both stochastic and structured input conditions. Specifically, $v_{t}$ is drawn from both smoothed random walks ${\tilde{v}}_{t}$ and deterministic regimes $u_{t}$ such as linear ramps and fixed-velocity intervals. The full $v_{t}$ is constructed as:

$$v_{t+1}=v_{t}+{\tilde{v}}_{t}+u_{t}$$ (1)

$${\tilde{v}}_{t+1}=0.8{\tilde{v}}_{t}+\frac{\alpha }{m}\left(\eta_{t}+\beta {\tilde{v}}_{t}\right),$$ (2)

where $\eta_{t}∼U\left(-1,1\right),\alpha ∼U\left(0,2\right),m∼U\left(10,625\right),\beta ∼U\left(0.05,0.45\right)$ with $v_{t}$ values clipped to [−1,1] to preserve realistic, bounded motion dynamics. This mixture of inputs produces rich and biologically plausible trajectories, challenging the neural network to generalize continuous integration across varied conditions.

**FIGURE 1:**
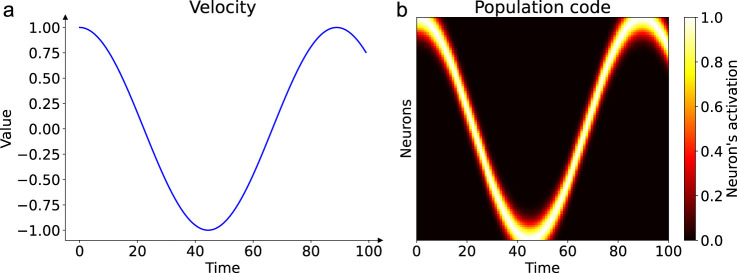
Population coding of a dynamic input signal. Panel **(a)** shows an example of a time-varying velocity signal, here one period of a sinusoid, presented to the network over time. Panel **(b)** illustrates how this scalar profile is encoded as a spatiotemporal activity pattern across the input neuron population. Each neuron (y-axis) is tuned to a preferred velocity. As the input velocity changes over time (x-axis), the peak of the activity bump shifts across the population, creating a dynamic representation of the signal shown in **(a)**.

To mimic the manner in which biological circuits represent continuous variables, we employ population coding for the input: the scalar velocity $v_{t}$ is encoded as a distributed neural activation pattern $I_{t}\in R^{n}$ , with each neuron having a Gaussian tuning curve centered on a specific preferred velocity value. As illustrated in Figure 1, this encoding transforms a dynamic, time-varying scalar signal into a spatiotemporal pattern of activity across the input population, which then serves as the input to the recurrent network:

$$P:\;v_{t}\to I_{t}\in R^{n}.$$ (3)

Such population codes enable robust integration and flexible transformation of noisy or ambiguous sensory signals, paralleling mechanisms observed in the brain’s sensory and motor systems. Notably, our input population encodes only velocity, not angular position, which differs from some biological head-direction systems where conjunctive coding is observed (Yoder et al., 2015). This choice allows us to specifically investigate how a network can autonomously learn to transform a pure velocity signal into a stable angular representation through its recurrent dynamics.

The target coordinate at time t , denoted $x_{t}$ , reflects the agent’s true angular position as obtained by integrating the velocity input:

$$x_{t}=\left(\int_{0}^{t}v_{\tau }\;d\tau \right)\;\mathrm{mod}\;2\pi .$$ (4)

In our discrete-time simulation, this integral is approximated using a second-order Euler method. During training, this serves as the supervisory signal. The “target neuron” refers to the output neuron whose preferred position ${\tilde{x}}_{n}$ is closest to $x_{t}$ at each step, a construct central to defining the network loss. We acknowledge that the use of an external, ground-truth supervisory signal is a simplification. In a biological context, such a signal would not be explicitly available. However, it can be interpreted as an abstraction of learning guided by other sensory modalities (e.g., visual landmarks) or by corrective feedback loops during development and exploration (Yoder et al., 2015; Levenstein et al., 2024). This supervised framework, also used in similar computational studies (Cueva and Wei, 2018), allows us to efficiently probe the types of network architectures and dynamics that are effective solutions for the task, revealing principles of self-organization that may be achieved through more implicit, biologically plausible learning mechanisms.

We arrange the neurons of the input and output populations to uniformly tile the relevant ranges, [−1,1] and $\left.\left[0,2\pi \right.\right)$ respectively, defining each neuron’s preferred value by $\tilde{x}$ . Population codes prevent the angular discontinuity that would otherwise occur at the 2π boundary, providing continuity for the network’s internal representations—a property essential for handling circular variables.

**FIGURE 2:**
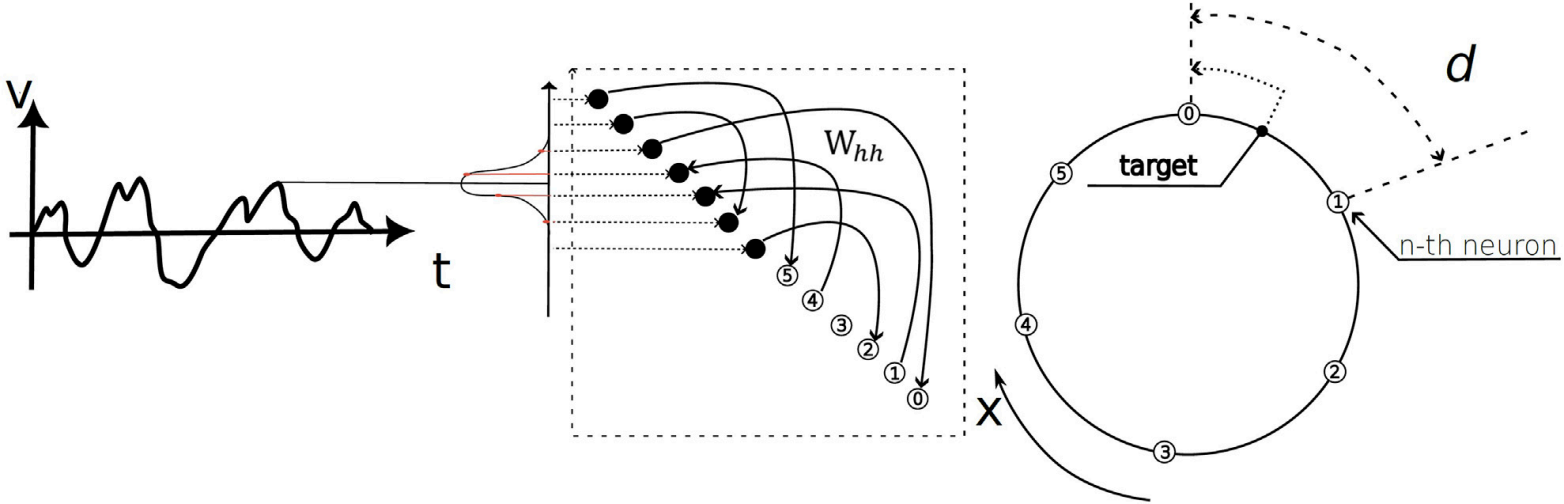
Schematic of the ring navigation task and network architecture. The model performs path integration by processing a time-varying velocity profile, V(t) , as input (left panel). The network architecture (middle panel) consists of a recurrent layer with two functionally distinct populations: an input population that encodes the incoming velocity and an output population that integrates this signal to produce an activity pattern representing the angular coordinate. Both populations are coupled through the recurrent weight matrix, $W_{hh}$ . The output neurons represent a circular coordinate space, x (right panel). During training, the network learns to align its activity with a target coordinate. The parameter d represents the minimal angular distance between the preferred positions of adjacent neurons, defining the spatial resolution of the population code.

The overall task and network architecture, which consists of functionally distinct input and output populations coupled through a recurrent weight matrix, is schematized in Figure 2. The artificial recurrent neural network we trained evolves according to:

$$h_{t+1}=\text{ReLU}\left(W_{hh}h_{t}+I_{t}\right),$$ (5)

where $W_{hh}$ is the recurrent weight matrix. We employ the ReLU activation function due to its computational efficiency and qualitative biological plausibility (Andreevic et al., 2025). While our model utilizes non-spiking neurons to maintain computational tractability, its emergent dynamics offer insights into the functional organization that may be implemented by more biophysically detailed, spiking networks (Song and Wang, 2005). Network stability is supported by orthogonal initialization, gradient clipping, and a curriculum learning protocol that increases trial length over training. The weights are optimized with Adam gradient descent using a population-based error function (loss) described below.

Our loss function is designed to reflect the graded, population-level readout present in biological codes. The neuron nearest to the target coordinate is treated as the “class” with the correct label, with adjacent neurons given smaller weights:

$$L=\gamma \overset{T}{\underset{t=1}{\sum }}\overset{N}{\underset{n=1}{\sum }}h_{t,n}-\frac{1}{T}\overset{T}{\underset{t=1}{\sum }}{\left(1+\frac{t}{T}\right)}^{\alpha }\overset{N/2}{\underset{n=1}{\sum }}\omega_{t,n}o_{t,n},$$ (6)

with

$$o_{t,n}=\log\left(\frac{\exp\left(h_{t,n}\right)}{\sum_{n^{\prime }=1}^{N}\exp\left(h_{t,n^{\prime }}\right)}\right),$$ (7)

and

$$\omega_{\mathrm{t,n}}=\frac{\exp\left(d_{t,n}\right)}{\sum_{n^{\prime }=1}^{N}\exp\left(d_{t,n^{\prime }}\right)},$$ (8)

where $d_{t,n}$ is the minimal circular distance between the target position $x_{t}$ and the preferred position ${\tilde{x}}_{n}$ of the n -th output neuron. The hyperparameter α accentuates the importance of accurate integration later in the trial, while γ regularizes total network activity.

To prevent units in the output population from becoming permanently silent (a common issue in ReLU networks), we implemented a simple homeostatic mechanism. In addition to adding a small, loss-adaptive noise to the gradients during each training step, we also periodically reinitialized inactive neurons. At the end of several batches, any neuron whose activity remained below a small threshold $\left(\epsilon =10^{-6}\right)$ for the entire trial duration was considered inactive. The weights of these inactive neurons are periodically reinitialized (using the average of their neighbors’ weights) and their optimizer state reset. This is analogous to homeostatic regulation in biological circuits and ensures all computational resources are utilized.^1^

## 3 Results

We designed our network with a pre-defined modular structure to investigate functional specialization. The total population of N = 800 neurons was partitioned into two equally sized groups: the first 400 neurons were designated as the “output population” and received no direct external input, while the remaining 400 neurons were the “input population” and received the external velocity signal. Both populations were uniformly tiled over their respective domains, creating a structured basis for analysis of functional specialization and connectivity.

To dissect the computational dynamics, we express the network’s full state at time t as the concatenation of input and output population activities:

$$h_{t}=\left[\begin{array}{c} h_{t}^{o}\\ h_{t}^{i} \end{array}\right]$$ (9)

with $h_{t}^{i}$ and $h_{t}^{o}$ denoting the activities of input and output populations. The recurrent weight matrix $W_{hh}$ naturally decomposes as:

$$W_{hh}=\left(\begin{array}{cc} W_{oo} & W_{oi}\\ W_{io} & W_{ii} \end{array}\right)$$ (10)

corresponding to intra-population (diagonal blocks $W_{oo},W_{ii}$ ) and inter-population (off-diagonal blocks $W_{oi},W_{io}$ ) connectivity.

The evolution of each sub-population is governed by:

$$h_{t+1}^{i}=\text{ReLU}\left(W_{ii}h_{t}^{i}+W_{io}h_{t}^{o}+I_{t}\right)$$ (11)

$$h_{t+1}^{o}=\text{ReLU}\left(W_{oo}h_{t}^{o}+W_{oi}h_{t}^{i}\right)$$ (12)

which capture the continuous integration, stabilization, and transformation of sensory input in a modular, population-based format.

**FIGURE 3:**
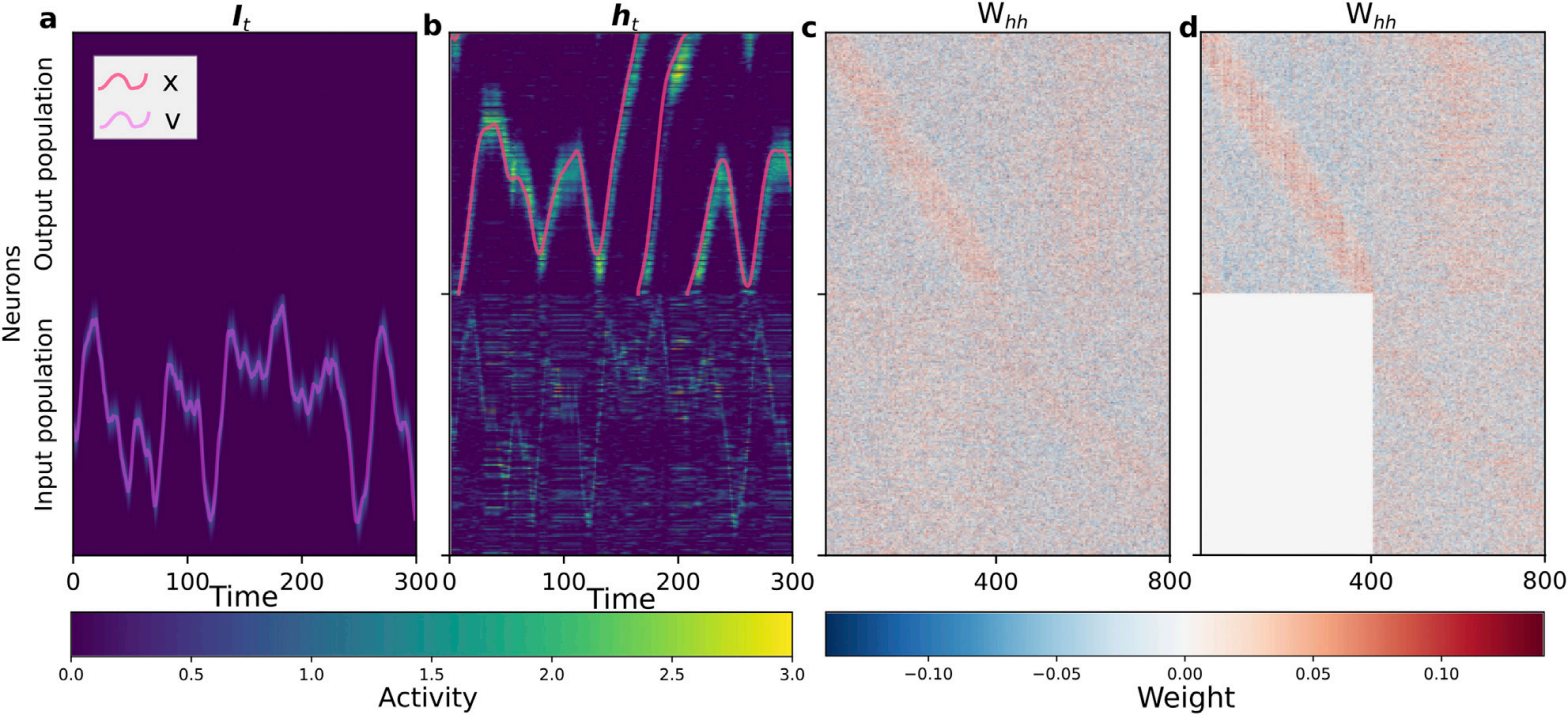
Self-organized network structure and dynamics during path integration. The network consists of an input population (neurons 1–400) and an output population (neurons 401–800). **(a)** Spatiotemporal activity of the population-coded input signal $\left(I_{t}\right)$ fed to the network, with the original scalar velocity profile superimposed (purple curve). **(b)** The resulting spatiotemporal activity $\left(h_{t}\right)$ of the fully trained recurrent network, with the decoded output coordinate superimposed (red curve). The output population (top half) maintains a persistent bump of activity that integrates the input, while the input population (bottom half) dynamically reflects the signal shown in **(a)**. **(c)** The learned recurrent weight matrix $\left(W_{hh}\right)$ from the fully trained network. The matrix reveals a clear self-organized modular structure, where the output-to-output block ( $W_{oo}$ , top-left) has formed a ring attractor with strong weights along the main diagonal. **(d)** The learned recurrent weight matrix $\left(W_{hh}\right)$ from a network that was trained with the feedback block from output to input $\left(W_{io}\right)$ permanently nullified.

A successfully trained network exhibits clear functional specialization, as shown in Figure 3. While the input population activity directly mirrors the incoming velocity signal (Figure 3a), the output population integrates this signal to maintain a stable, localized bump of activity representing the agent’s angular position (Figure 3b). This functional division is a direct result of the self-organized recurrent connectivity matrix (Figure 3c), where the output-to-output block $\left(W_{oo}\right)$ develops a robust ring-shaped attractor structure along its diagonal, which enables stable representations of position. We use the term ‘attractor’ to describe the functional behavior of the network, which creates quasi-stable states that are robust to noise and persist over timescales relevant for the task, even if they are not attractors in the strict mathematical sense of having infinite stability. In contrast, the input population’s recurrent weights $\left(W_{ii}\right)$ show a more localized structure. These learned weights facilitate, respectively, the persistence of spatial memory and the rapid encoding of transient velocity inputs.

To further probe the specialization of network modules, we simulated the autonomous activity of the input and output populations in the absence of inter-population coupling. Here, each population was initialized with Gaussian bumps at multiple, distinct positions. This setup allows us to differentiate persistent from transient attractor dynamics. In this isolated condition, the activity of the input $\left({\tilde{h}}^{i}\right)$ and output $\left({\tilde{h}}^{o}\right)$ populations evolves according to:

$${\tilde{h}}_{t+1}^{i}=\text{ReLU}\left(W_{ii}{\tilde{h}}_{t}^{i}\right)$$ (13)

$${\tilde{h}}_{t+1}^{o}=\text{ReLU}\left(W_{oo}{\tilde{h}}_{t}^{o}\right)$$ (14)

**FIGURE 4:**
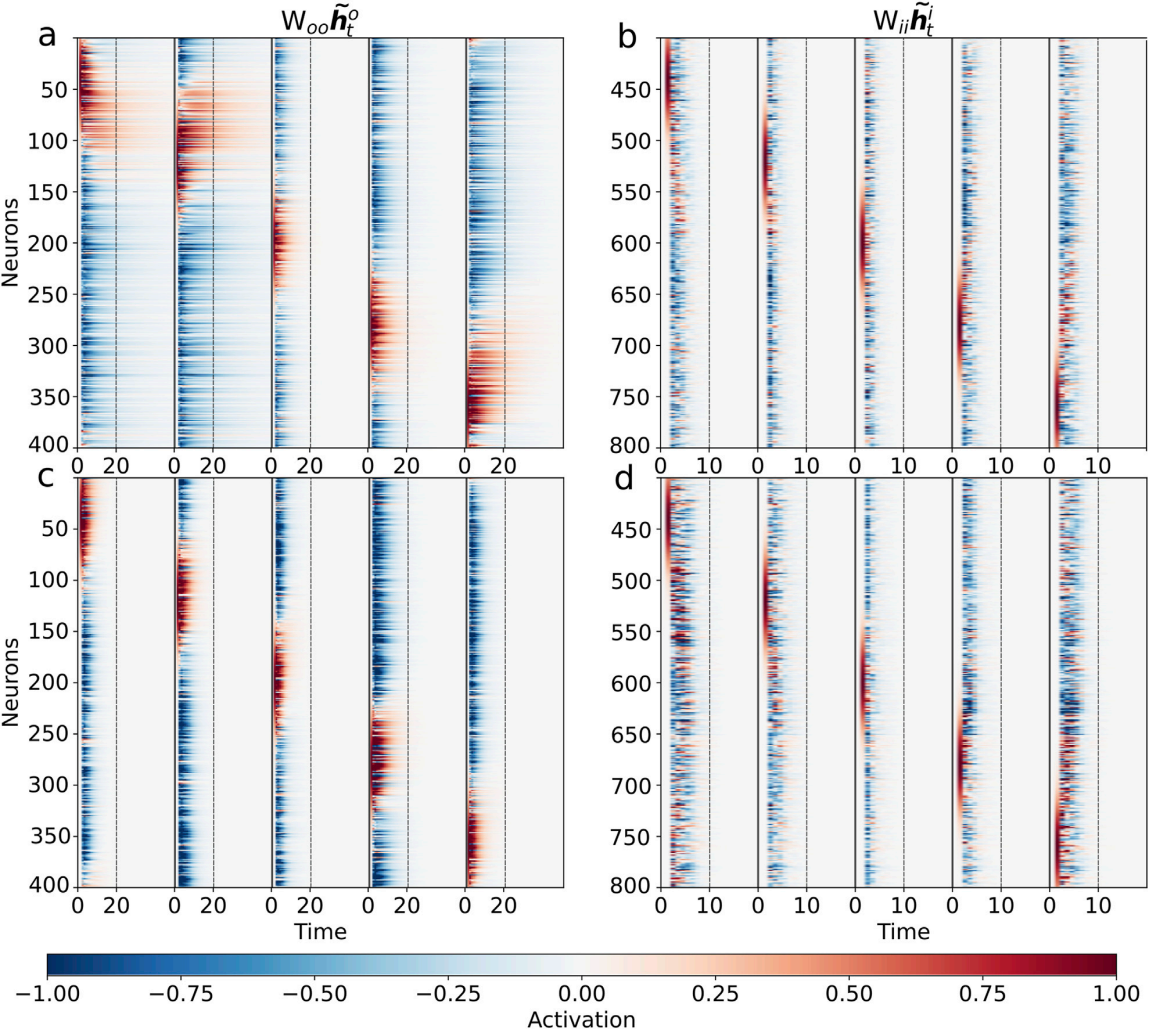
Autonomous dynamics reveal functional specialization of input and output populations. To test the intrinsic properties of the learned modules, we simulated their autonomous activity driven only by their internal recurrent connections (i.e., without external input or inter-population signals). Each population was initialized with five distinct Gaussian bumps of activity. **(a,c)** The output populations exhibit transiently persistent activity, maintaining the bumps for an extended period before eventually decaying to the zero state. This demonstrates a clear memory-like capability, essential for integration. Notably, the persistence in the fully connected network **(a)** is significantly more robust, sustaining the localized activity much longer than in the feedforward-only network **(c)**. **(b,d)** In sharp contrast, the input populations from both the fully connected network **(b)** and the feedforward-only network **(d)** show dissipative dynamics, where the initial activity bumps decay rapidly. These simulations correspond to evolving the activity according to (Pouget et al., 2000; Hafting et al., 2005).

The results of these autonomous simulations, shown in Figure 4, confirm the functional roles of the two modules. The output population sustains the initial activity bumps for an extended period (Figure 4a), demonstrating a memory-like capability. By contrast, the input population shows rapid decay (Figure 4b), consistent with its role as a transient encoder. We note that the specific network instance visualized here shows some heterogeneity in its response, with stronger activity in one region of the ring. This is a feature of this particular trained network, as the effect varies across different training runs and is not a systematic bias of the model. The central finding demonstrated here—the clear functional contrast between the persistent output and dissipative input populations—is a robust result observed consistently across all successful networks.

To investigate the role of modular connectivity, we selectively ablated the feedback pathway $\left(W_{io}\right)$ from the output to the input population. This manipulation tests the importance of top-down signals for stabilizing network dynamics. We observed that while the purely feedforward architecture could still perform integration to some degree, the activity in the output population became less stable and exhibited significant drift over time, leading to a rapid degradation in positional accuracy.

The underlying reason for this instability is revealed by comparing the autonomous dynamics of the two networks in Figure 4. The output module from the network trained without feedback is intrinsically less stable, showing a much faster decay of activity (Figure 4c) compared to its counterpart from the fully connected network (Figure 4a). This highlights that the network learns to sustain activity for a prolonged but finite duration, a property we term transient persistence. This suggests that in the full network, the feedback pathway allows the input population to participate in a larger recurrent circuit that actively stabilizes the activity bump on the output ring. Without this top-down connection, the entire burden of maintaining persistent activity falls solely upon the internal recurrence of the output population $\left(W_{oo}\right)$ , rendering the memory trace more susceptible to decay and noise. This demonstrates the critical role of the complete recurrent structure, including feedback loops, for robust memory maintenance.

We then explored the mapping and transfer of control signals by delivering a velocity input that increased linearly from −1 to 1. The signal propagation from input to output was visualized and further tested by permuting the ordering of output neurons (breaking the topological alignment between population code and ring structure).

**FIGURE 5:**
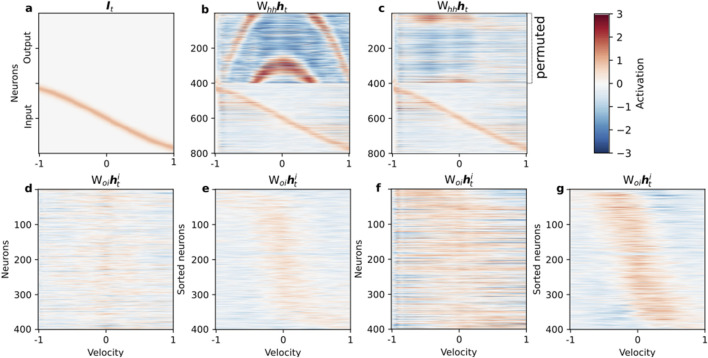
Topological alignment of control signals is critical for integration. We probed the network’s integration mechanism using a linearly increasing velocity input. **(a)** The input velocity ramp from −1 to 1 over the trial. **(b)** The activity of the fully trained network $\left(W_{hh}h_{t}\right)$ shows successful integration, evidenced by the smoothly moving bump in the output population (neurons 1–400). **(c)** However, randomly permuting the order of neurons within the output population disrupts the learned topological mapping. This single change completely abolishes integration, resulting in chaotic network activity. **(d–g)** Visualization of the feedforward control signal $\left(W_{oi}h_{t}^{i}\right)$ from the input to the output population, plotted as a function of the input velocity. **(d)** For the fully trained network, the raw control signal appears unstructured. **(e)** However, when the output neurons are sorted by their peak response velocity, a highly structured diagonal pattern emerges, revealing a precise, topographically organized ‘push’ that systematically drives the activity bump. **(f,g)** Remarkably, the same analysis for the network trained without feedback reveals a nearly identical underlying control structure **(g)** when sorted. This striking similarity demonstrates that the dynamic mechanism for controlling the coordinate is learned via the feedforward pathway in both networks. This supports the hypothesis that the feedforward projection $\left(W_{oi}\right)$ is responsible for driving the movement of the representation, while the recurrent connections within the output population $\left(W_{oo}\right)$ are primarily responsible for stabilizing it.

As illustrated in Figure 5b, a properly trained network successfully integrates the velocity ramp (Figure 5a). However, network performance collapses when the population code-to-ring mapping is disrupted by permuting the output neurons (Figure 5c), confirming that the encoded structure and network topology must remain aligned for successful integration. The output population in the unperturbed circuit receives a smoothly propagating population control signal, but this signal can no longer synchronize correctly with a shuffled arrangement, revealing the codependency of circuitry and coding motif.

Further analysis of the feedforward control signal $\left(W_{oi}h_{t}^{i}\right)$ provides a key insight into the network’s modular strategy. As shown in Figures 5d–g, while the raw control signal appears noisy (Figures 5d,f), sorting the output neurons by their peak response velocity reveals a highly organized diagonal structure in both the full network (Figure 5e) and the feedforward-only network (Figure 5g). The remarkable similarity between these two sorted signals strongly suggests that the fundamental mechanism for converting velocity into a directional “push” on the output population is implemented by the feedforward pathway $\left(W_{oi}\right)$ . This confirms that the role of the feedback pathway $\left(W_{io}\right)$ is not to shape the control signal itself, but rather to contribute to the overall dynamic stability of the network, a conclusion consistent with the autonomous dynamics shown in Figure 4.

Taken together, these analyses show that population-coded recurrent networks can naturally self-organize into specialized modules for fast encoding and persistent memory. Faithful function relies not only on the learned synaptic weights, but also on the precise and consistent internal mapping between neural populations and their target representations. These features are hallmarks of modular, robust physiological computation as observed in biological navigation and memory systems.

## 4 Discussion and conclusion

In this study, we demonstrated that a recurrent neural network trained on a continuous integration task can autonomously self-organize into a modular architecture with functionally distinct and physiologically congruent subpopulations. Here, self-organization refers to the emergence of structured connectivity as a result of a supervised learning process, rather than arising from unsupervised, local update rules in the physical sense. Our findings contribute to bridging the gap between the dynamics of artificial neural networks and the principles of neural computation observed in biological systems.

Our central finding is the emergent division of labor within the network. The output subpopulation develops a ring attractor, a canonical structure for encoding circular variables like head direction and spatial orientation (Zhang, , 1996; Kim et al., 2017). This structure supports persistent, localized activity, enabling it to function as a robust memory module for the integrated position. Concurrently, the input subpopulation forms a dissipative, segment-like architecture that acts as a transient control unit, transforming velocity signals into directional commands that drive the movement of the activity bump on the ring. This modular separation—separating memory from control—is a key organizational principle in the brain, allowing for flexible and robust computation (Salinas and Abbott, 1994; Ganguli and Sompolinsky, 2012). Unlike models where ring connectivity is hardwired, here it emerges solely from the learning objective, suggesting that attractor dynamics are a natural and efficient solution for continuous variable integration.

A key distinction of our work, however, is the demonstration of the critical importance of topological alignment between these emergent modules. As shown in our permutation experiment (Figures 5a–c), the network’s function is not merely a product of its component parts but depends fundamentally on the learned, ordered mapping between the control signals from the input population and the spatial layout of the output ring attractor. This highlights that for distributed neural codes to be computationally effective, the wiring” must respect the coding”. Disruptions to this alignment, analogous to developmental disorders or brain injury, can lead to a catastrophic failure of function, even if the individual modules remain intact. Our perturbation analyses thus underscore the role of feedback and precise inter-module connectivity, echoing experimental findings where disrupting specific pathways compromises memory and integration (Seeholzer et al., 2019; McNaughton et al., 2006; Bonnevie et al., 2013).

Our results also inform the broader field of network physiology by providing a concrete computational example of how specialized subsystems can arise and coordinate within a larger, interconnected system. The balance between the persistent dynamics of the memory module and the dissipative dynamics of the control module illustrates how networks can achieve both stability and adaptability. This emergent coordination within a complex neural network serves as a key example of physiological resilience and the principles of system-level self-organization that are central to the study of network physiology (Bartsch et al., 2015; Ivanov and Bartsch, 2014; Ivanov, 2021).

### 4.1 Limitations and future directions

We acknowledge several limitations that open avenues for future research. First, our model employs non-spiking neurons and a supervised, gradient-based learning rule. While this framework is computationally powerful, future work should explore how these functional architectures could emerge using more biologically plausible spiking neurons and local, Hebbian-like learning rules (Song and Wang, 2005; Levenstein et al., 2024; Pugavko et al., 2023; Maslennikov et al., 2024). Second, the supervisory signal, while justifiable as an abstraction, could be replaced with reinforcement learning or unsupervised learning objectives to better model autonomous discovery in biological agents (Banino et al., 2018; Ivanov et al., 2025). Furthermore, training the network on more complex and physiologically grounded velocity profiles, such as those derived from animal tracking data (Sargolini et al., 2006), could reveal how network solutions are shaped by naturalistic input statistics. Exploring the network’s resilience to transient perturbations, such as temporary loss of connectivity between modules (Cooper and Mizumori, 2001), would also provide deeper insights into the robustness of these self-organized circuits.

On a translational level, our work illustrates how interpretable, modular architectures can be learned rather than handcrafted, offering a path toward more explainable AI and robust autonomous systems. This is particularly relevant for neuromorphic engineering and robotics, where many existing applications of ring attractors rely on hand-crafted weights (Rivero-Ortega et al., 2023). Our approach, where functional weights are learned, offers a promising route to developing more adaptive and flexible controllers. For mobile and field robotics, key considerations include not only robustness and interpretability but also low power consumption, a primary goal of neuromorphic systems (Izzo et al., 2023; Robin et al., 2022). Furthermore, the ring attractor motif is not limited to 1D orientation but serves as a foundational component for more complex spatial representations, such as modeling the 2D planar motion of a robot, linking back to the principles of grid cell computation (Knowles et al., 2023). The increasing availability of specialized neuromorphic hardware, such as Intel’s Loihi processors (Davies et al., 2018), and associated software frameworks like LAVA (Lava Framework Authors, 2022), makes these brain-inspired models increasingly viable for real-world, embedded applications where online learning and energy efficiency are paramount.

### 4.2 Conclusion

In summary, we have shown that explicit population coding guides a recurrent network to self-organize into a modular system comprising a ring attractor for memory and a dissipative controller for input processing. This emergent structure, highly reminiscent of biological circuits for navigation, depends critically on the precise topological alignment between its functional modules. Our findings underscore how general learning principles can give rise to specialized, interpretable, and physiologically plausible neural computations, advancing our understanding of both natural and artificial intelligence.

## Data Availability

The original contributions presented in the study are included in the article/supplementary material, further inquiries can be directed to the corresponding author.

## Footnotes

1 The code used to generate the results in this study is available at https://github.com/p0mik13/PopulCoding_RingNavigationTask_F.

## References

[B2] Averbeck B. B. Latham P. E. Pouget A. (2006). Neural correlations, population coding and computation. Nat. Rev. Neurosci. 7 (5), 358–366. 10.1038/nrn1888 16760916

[B3] BaninoA.BarryC.UriaB.BlundellC.LillicrapT.MirowskiP. (2018). Vector-based navigation using grid-like representations in artificial agents. Nature 557 (7705), 429–433. 10.1038/s41586-018-0102-6 29743670

[B4] Bartsch R. P. Liu K. K. L. Bashan A. Ivanov P.Ch (2015). Network physiology: how organ systems dynamically interact. PloS One 10 (11), e0142143. 10.1371/journal.pone.0142143 26555073 PMC4640580

[B5] Basu J. Nagel K. (2024). Neural circuits for goal-directed navigation across species. Trends Neurosci. 47 (11), 904–917. 10.1016/j.tins.2024.09.005 39393938 PMC11563880

[B6] Ben-Yishay E. Krivoruchko K. Ron S. Ulanovsky N. Derdikman D. Gutfreund Y. (2021). Directional tuning in the hippocampal formation of birds. Curr. Biol. 31 (12), 2592–2602.e4. 10.1016/j.cub.2021.04.029 33974847

[B7] Bonnevie T. Dunn B. Fyhn M. Hafting T. Derdikman D. Kubie J. L. (2013). Grid cells require excitatory drive from the hippocampus. Nat. Neurosci. 16 (3), 309–317. 10.1038/nn.3311 23334581

[B8] Bush D. Burgess N. (2014). A hybrid oscillatory interference/continuous attractor network model of grid cell firing. J. Neurosci. 34 (14), 5065–5079. 10.1523/JNEUROSCI.4017-13.2014 24695724 PMC3972729

[B9] Cooper B. G. Mizumori S. J. Y. (2001). Temporary inactivation of the retrosplenial cortex causes a transient reorganization of spatial coding in the hippocampus. J. Neurosci. 21 (11), 3986–4001. 10.1523/JNEUROSCI.21-11-03986.2001 11356886 PMC6762703

[B10] Cueva C. J. Wei X.-X. (2018). Emergence of grid-like representations by training recurrent neural networks to perform spatial localization. arXiv Prepr. arXiv:1803.07770. 10.48550/arXiv.1803.07770

[B11] Davies M. Srinivasa N. Lin T.-H. Chinya G. Cao Y. Choday S. H. (2018). Loihi: a neuromorphic manycore processor with on-chip learning. Ieee Micro 38 (1), 82–99. 10.1109/mm.2018.112130359

[B12] Fiete I. R. Burak Y. Brookings T. (2008). What grid cells convey about rat location. J. Neurosci. 28 (27), 6858–6871. 10.1523/JNEUROSCI.5684-07.2008 18596161 PMC6670990

[B13] Ganguli S. Sompolinsky H. (2012). Compressed sensing, sparsity, and dimensionality in neuronal information processing and data analysis. Annu. Rev. Neurosci. 35 (1), 485–508. 10.1146/annurev-neuro-062111-150410 22483042

[B14] Georgopoulos A. P. Schwartz A. B. Kettner R. E. (1986). Neuronal population coding of movement direction. Science 233 (4771), 1416–1419. 10.1126/science.3749885 3749885

[B15] Hafting T. Fyhn M. Molden S. Moser M.-B. Moser E. I. (2005). Microstructure of a spatial map in the entorhinal cortex. Nature 436 (7052), 801–806. 10.1038/nature03721 15965463

[B16] Haken H. (1983). Synergetics: an introduction. 3rd edition. Berlin: Springer.

[B17] Heinze S. Narendra A. Cheung A. (2018). Principles of insect path integration. Curr. Biol. 28 (17), R1043–R1058. 10.1016/j.cub.2018.04.058 30205054 PMC6462409

[B18] Ivanov P.Ch. (2021). The new field of network physiology: building the human physiolome. Front. Netw. physiology 1, 711778. 10.3389/fnetp.2021.711778 36925582 PMC10013018

[B19] Ivanov P.Ch Bartsch R. P. (2014). “Network physiology: mapping interactions between networks of physiologic networks,” in Networks of networks: the last frontier of complexity (Springer), 203–222.

[B20] Ivanov D. A. Larionov D. A. Maslennikov O. V. Voevodin V. V. (2025). Neural network compression for reinforcement learning tasks. Sci. Rep. 15 (1), 9718. 10.1038/s41598-025-93955-w 40118986 PMC11928627

[B21] Izzo D. Hadjiivanov A. Dold D. Meoni G. Blazquez E. (2023). “Neuromorphic computing and sensing in space,” in Artificial intelligence for space: AI4SPACE (Boca Raton, FL: CRC Press), 107–159. 10.48550/arXiv.2212.05236

[B22] Khona M. Fiete I. R. (2022). Attractor and integrator networks in the brain. Nat. Rev. Neurosci. 23 (12), 744–766. 10.1038/s41583-022-00642-0 36329249

[B23] Kim S. S. Rouault H. Druckmann S. Jayaraman V. (2017). Ring attractor dynamics in the drosophila central brain. Science 356 (6340), 849–853. 10.1126/science.aal4835 28473639

[B24] Knierim J. J. Kudrimoti H. S. McNaughton B. L. (1995). Place cells, head direction cells, and the learning of landmark stability. J. Neurosci. 15 (3), 1648–1659. 10.1523/JNEUROSCI.15-03-01648.1995 7891125 PMC6578145

[B25] Knowles T. C. Summerton A. G. Whiting J. G. H. Pearson M. J. (2023). Ring attractors as the basis of a biomimetic navigation system. Biomimetics 8 (5), 399. 10.3390/biomimetics8050399 37754150 PMC10526409

[B1] Kononov R. A. Maslennikov O. V. Nekorkin V. I. (2025). Dynamics of recurrent neural networks with piecewise linear activation function in the context-dependent decision-making task. Izv. VUZ. Appl. Nonlinear Dyn. 33 (2), 249–265. 10.18500/0869-6632-003147

[B26] Lava Framework Authors (2022). Lava: a framework for neuromorphic computing.

[B27] Levenstein D. Efremov A. Eyono R. H. Peyrache A. Richards B. (2024). Sequential predictive learning is a unifying theory for hippocampal representation and replay. bioRxiv, 2024–04. 10.1101/2024.04.28.591528

[B28] Maslennikov O. Perc M. Nekorkin V. (2024). Topological features of spike trains in recurrent spiking neural networks that are trained to generate spatiotemporal patterns. Front. Comput. Neurosci. 18, 1363514. 10.3389/fncom.2024.1363514 38463243 PMC10920356

[B41] Maslennikov O. V. Pugavko M. M. Shchapin D. S. Nekorkin V. I. (2022). Nonlinear dynamics and machine learning of recurrent spiking neural networks. Physics-Uspekhi 65 (10), 1020–1038. 10.3367/ufne.2021.08.039042

[B29] McNaughton B. L. Battaglia F. P. Jensen O. Moser E. I. Moser M.-B. (2006). Path integration and the neural basis of the’cognitive map. Nat. Rev. Neurosci. 7 (8), 663–678. 10.1038/nrn1932 16858394

[B30] Moser E. I. Kropff E. Moser M. B. (2008). Place cells, grid cells, and the brain's spatial representation system. Annu. Rev. Neurosci. 31, 69–89. 10.1146/annurev.neuro.31.061307.090723 18284371

[B31] Pouget A. Dayan P. Zemel R. S. (2000). Information processing with population codes. Nat. Rev. Neurosci. 1 (2), 125–132. 10.1038/35039062 11252775

[B32] Pugavko M. M. Maslennikov O. V. Nekorkin V. I. (2023). Multitask computation through dynamics in recurrent spiking neural networks. Sci. Rep. 13 (1), 3997. 10.1038/s41598-023-31110-z 36899052 PMC10006454

[B33] Rivero-Ortega J. D. Mosquera-Maturana J. S. Pardo-Cabrera J. Hurtado-López J. Hernández J. D. Romero-Cano V. (2023). Ring attractor bio-inspired neural network for social robot navigation. Front. Neurorobotics 17, 1211570. 10.3389/fnbot.2023.1211570 37719331 PMC10501606

[B34] Robinson B. S. Norman-Tenazas R. Cervantes M. Symonette D. Johnson E. C. Joyce J. (2022). Online learning for orientation estimation during translation in an insect ring attractor network. Sci. Rep. 12 (1), 3210. 10.1038/s41598-022-05798-4 35217679 PMC8881593

[B35] Salinas E. Abbott L. F. (1994). Vector reconstruction from firing rates. J. Comput. Neurosci. 1 (1), 89–107. 10.1007/BF00962720 8792227

[B36] Sargolini F. Fyhn M. Hafting T. McNaughton B. L. Witter M. P. Moser M.-B. (2006). Conjunctive representation of position, direction, and velocity in entorhinal cortex. Science 312 (5774), 758–762. 10.1126/science.1125572 16675704

[B37] Seeholzer A. Moritz D. Gerstner W. (2019). Stability of working memory in continuous attractor networks under the control of short-term plasticity. PLoS Comput. Biol. 15 (4), e1006928. 10.1371/journal.pcbi.1006928 31002672 PMC6493776

[B38] Skaggs W. Knierim J. Kudrimoti H. McNaughton B. (1994). A model of the neural basis of the rat’s sense of direction. Adv. Neural Inf. Process. Syst. 7. Available online at: https://pubmed.ncbi.nlm.nih.gov/11539168/ . 11539168

[B39] Song P. Wang X.-J. (2005). Angular path integration by moving “hill of activity”: a spiking neuron model without recurrent excitation of the head-direction system. J. Neurosci. 25 (4), 1002–1014. 10.1523/JNEUROSCI.4172-04.2005 15673682 PMC6725619

[B40] Vinepinsky E. Cohen L. Perchik S. Ben-Shahar O. Donchin O. Segev R. (2020). Representation of edges, head direction, and swimming kinematics in the brain of freely-navigating fish. Sci. Rep. 10 (1), 14762. 10.1038/s41598-020-71217-1 32901058 PMC7479115

[B42] Yoder R. M. Peck J. R. Taube J. S. (2015). Visual landmark information gains control of the head direction signal at the lateral mammillary nuclei. J. Neurosci. 35 (4), 1354–1367. 10.1523/JNEUROSCI.1418-14.2015 25632114 PMC4308588

[B43] Zhang K. (1996). Representation of spatial orientation by the intrinsic dynamics of the head-direction cell ensemble: a theory. J. Neurosci. 16 (6), 2112–2126. 10.1523/JNEUROSCI.16-06-02112.1996 8604055 PMC6578512

